# Selective lymph node dissection in intrahepatic cholangiocarcinoma and combined hepatocellular cholangiocarcinoma may not impair oncological outcomes: a single-center retrospective cohort study

**DOI:** 10.1186/s12957-025-04034-3

**Published:** 2025-10-21

**Authors:** Wei-Hsun Lu, Ting-Kai Liao, Che-Min Su, Tsung-Han Yang, Tsung-Ching Chou, Ping-Jui Su, Chih-Jung Wang, Ying Jui Chao, Yih-Jyh Lin, Yan-Shen Shan

**Affiliations:** 1 Department of Surgery, College of Medicine, National Cheng Kung University Hospital, National Cheng Kung University, Tainan, Taiwan; 2 Institute of Clinical Medicine, College of Medicine, National Cheng Kung University, Tainan, Taiwan; 3 Department of Biomedical Engineering, The International Institute of Medical Device Innovation, National Cheng Kung University, Tainan, Taiwan

**Keywords:** Intrahepatic cholangiocarcinoma, Combined hepatocellular cholangiocarcinoma, Lymph node dissection, Hepatectomy, Survival analysis, Surgical oncology

## Abstract

**Background:**

Current guidelines recommend routine lymph node dissection (LND) for intrahepatic cholangiocarcinoma (iCCA) to achieve adequate staging; however, real-world compliance remains suboptimal. This study evaluated whether, compared with routine approaches, selective lymphadenectomy, on the basis of clinical judgment, compromises oncological outcomes in patients with iCCA.

**Methods:**

A retrospective analysis of 179 patients who underwent curative hepatectomy for iCCA between 2014 and 2024 was performed. The cohort included pure cholangiocarcinoma (CCA, *n* = 102) and combined hepatocellular-cholangiocarcinoma (HCC-CCA, *n* = 77) patients. Patients were categorized by pathological nodal status: pN0 (LND performed, negative nodes), pN1 (LND performed, positive nodes), and pNx (no LND performed). Logistic regression identified factors influencing LND decisions. Survival outcomes were analyzed via the Kaplan‒Meier method and Cox proportional hazards modeling. Subgroup analysis was performed to explore the outcomes in CCA and HCC-CCA separately.

**Results:**

LND was performed in 54 patients (30%), with significant variation based on tumor characteristics. Preoperative cholangiocarcinoma diagnosis was the primary factor influencing LND decisions (OR 3.33, 95% CI 1.54–7.34; *p* = 0.002). The median overall survival (OS) was 30.5, 17.4, and 59.1 months (*p* = 0.007), and median progression-free survival (PFS) was 21.2, 8.4, and 16.6 months (*p* = 0.042) for pN0, pN1, and pNx, respectively. Subgroup analysis for CCA and HCC-CCA separately showed a similar Kaplan-Meier curve pattern, but the differences were not statistically significant because of the uneven distribution between groups. After adjusting for age, tumor stage, and histology, no significant difference in survival was detected between the pNx and pN0 groups (HR 0.78, 95% CI 0.46–1.30; *p* = 0.34). Patients with pure CCA had worse survival than those with HCC-CCA (HR 1.68, 95% CI 1.03–2.75; *p* = 0.040). Adequate lymphadenectomy (≥ 6 nodes) was achieved in only 26% of patients who underwent LND.

**Conclusions:**

This study highlights the low compliance with the guidelines regarding lymph node dissection for intrahepatic cholangiocarcinoma in real-world settings. However, compared to lymphadenectomy with negative nodes, selective lymph node dissection based on clinical suspicion does not compromise the overall survival. These findings support individualized surgical approaches rather than universal lymphadenectomy protocols and challenge current guidelines mandating routine LND for all iCCA patients. Future guidelines should incorporate risk-stratified decision-making in lymph node management.

**Supplementary Information:**

The online version contains supplementary material available at 10.1186/s12957-025-04034-3.

## Introduction

Intrahepatic cholangiocarcinoma (iCCA) is a primary liver malignancy that includes pure cholangiocarcinoma (CCA) and combined hepatocellular cholangiocarcinoma (HCC-CCA). The global incidence of iCCA has increased substantially over recent decades, with reported increases of up to 165% in some regions [1, 2]. This increasing incidence has been attributed to various risk factors, including hepatitis B and C infections, liver cirrhosis, primary sclerosing cholangitis, and exposure to carcinogens, with significant geographic variations in risk factor prevalence [3, 4]. Epidemiological patterns differ markedly between Eastern and Western populations, with viral hepatitis being more prevalent in Asian countries and primary sclerosing cholangitis predominating in Western nations [4].

Surgical resection remains the only potentially curative treatment for iCCA [5, 6]. However, iCCA represents a heterogeneous group of malignancies with distinct molecular characteristics and biological behaviors [7, 8]. The genetic landscape of tumors is characterized by significant heterogeneity, presenting major challenges for targeted therapies and personalized treatment approaches [7]. Recent advances in understanding molecular pathogenesis have revealed multiple oncogenic pathways and potential therapeutic targets, opening new horizons for precision medicine in cholangiocarcinoma management [8].

Combined hepatocellular cholangiocarcinoma (HCC-CCA) presents unique diagnostic and therapeutic challenges because of its dual cellular characteristics [9–12]. This rare tumor type, comprising both hepatocytic and cholangiocytic components, has been subject to evolving nomenclature and diagnostic criteria [12]. Recent studies suggest that combined HCC-CCA may have different prognostic implications than pure cholangiocarcinoma does, with some reports indicating potentially better survival outcomes [9, 11]. The accurate diagnosis and optimal management of these mixed tumors remain areas of active investigation [10].

Current clinical practice guidelines recommend routine lymph node dissection for iCCA, suggesting that adequate lymphadenectomy should include the assessment of regional lymph nodes to harvest at least six lymph nodes for proper staging [13–16]. These recommendations are based on the premise that lymph node status is a critical prognostic factor and that adequate staging is essential for optimal patient management [17]. However, compliance with these recommendations remains suboptimal in real-world practice. Recent studies have demonstrated that adequate lymphadenectomy (harvesting ≥ 6 lymph nodes) is performed in only 10–17% of iCCA patients [18, 19]. This discrepancy between guideline recommendations and clinical practice raises essential questions about the practical applicability and necessity of routine lymphadenectomy for all patients with iCCA.

The therapeutic landscape for iCCA has evolved significantly, with advances in both surgical techniques and systemic therapies. While surgical resection remains the cornerstone of curative treatment, the role of adjuvant chemotherapy has been established, with studies demonstrating survival benefits [20, 21]. Recent developments in immunotherapy have also shown promise, offering new treatment options for advanced disease [22]. These therapeutic advances underscore the importance of accurate staging and patient selection for optimal treatment outcomes.

This study aims to evaluate real-world practice patterns in lymph node management for iCCA and assess whether selective lymphadenectomy approaches compromise oncological outcomes.

## Methods

### Study design and patient selection

**Fig. 1:**
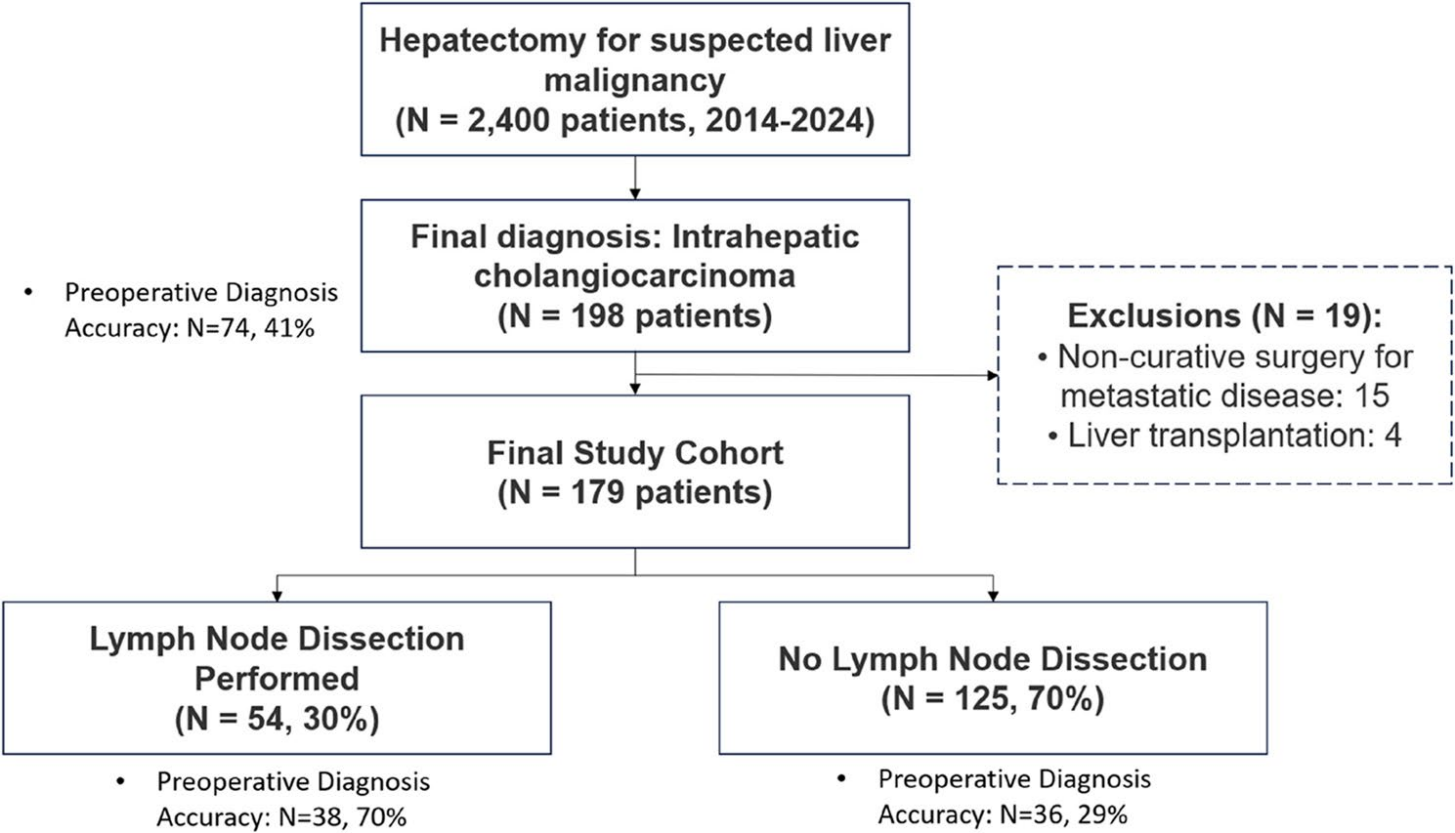
Patient flow diagram for an intrahepatic cholangiocarcinoma study, illustrating the patient selection process and distribution by lymphadenectomy status

This was a retrospective study from a single medical center. The patients who underwent hepatectomy at our institution between 2014 and 2024 were included. Among the 2,400 hepatectomies performed during this period, 198 patients had a final diagnosis of intrahepatic cholangiocarcinoma. We excluded 19 patients: 15 who underwent noncurative surgery for metastatic disease and four who underwent liver transplantation. The final study cohort comprised 179 patients with histologically confirmed intrahepatic cholangiocarcinoma (iCCA), including pure cholangiocarcinoma (CCA, *n* = 102) and combined hepatocellular-cholangiocarcinoma (HCC-CCA, *n* = 77) (Fig. 1).

## Institutional lymph node dissection strategy

At our institution, lymph node dissection decisions were made intraoperatively based on the attending surgeon’s clinical judgment. The primary factors influencing LND decisions included: (1) preoperative diagnostic confidence of cholangiocarcinoma based on imaging characteristics, tumor markers, and clinical presentation; (2) intraoperative tumor appearance and characteristics; (3) tumor location (perihilar vs. peripheral); and (4) patient comorbidities and operative risk assessment—no standardized protocol mandated routine lymphadenectomy for all patients. Surgeons typically performed LND when there was high clinical suspicion of cholangiocarcinoma, particularly for perihilar tumors or when imaging suggested nodal involvement. For patients with suspected hepatocellular carcinoma or when the diagnosis was uncertain, LND was often omitted to minimize operative morbidity.

## Data collection

Comprehensive clinicopathological data, including patient demographics, preoperative characteristics, operative details, pathological findings, and follow-up outcomes, were collected from medical records. Preoperative cholangiocarcinoma diagnosis was defined as clinical suspicion of cholangiocarcinoma on the basis of imaging, tumor markers, and clinical presentation before surgery.

Lymph node management was categorized on the basis of pathological nodal status: pN0 (lymphadenectomy performed with negative nodes), pN1 (lymphadenectomy performed with positive nodes), and pNx (no lymphadenectomy performed). Adequate lymphadenectomy was defined as the retrieval of 6 or more lymph nodes. The tumor location was classified as perihilar (involving the hepatic hilum or major intrahepatic ducts) or peripheral according to the definition with the JSHBPS classification [23]. The resection margin status was classified according to the 1 mm rule: R0 (>1 mm clear margin), R1 (≤ 1 mm margin), or R2 (macroscopically positive margin).

### Statistical analysis

Categorical variables are presented as frequencies and percentages, whereas continuous variables are presented as medians with interquartile ranges. Comparisons between groups were performed via chi-square tests for categorical variables and Mann‒Whitney U tests for continuous variables.

Factors associated with lymphadenectomy decisions were analyzed via logistic regression. Variables with *p* < 0.10 in the univariable analysis were included in the multivariable modeling. Overall survival was calculated from the date of surgery to the date of death or the last follow-up. Survival curves were constructed via the Kaplan‒Meier method and compared via Peto-Peto tests and pairwise comparisons using log-rank tests with Bonferroni correction. Cox proportional hazards regression was used to identify independent prognostic factors for survival. Subgroup analysis separating CCA from HCC-CCA was performed to illustrate further the differential effect of lymph node dissection among these two histological types of tumors.

Statistical significance was set at *p* < 0.05. All analyses were performed via R statistical software (version 4.5.1).

## Results

### Patient and tumor characteristics

Among the 179 patients, LND was performed in 54 patients (30%). Notably, only 41% of the patients were diagnosed with cholangiocarcinoma prior to surgery. In the LND + group, the diagnostic accuracy rate was as high as 70%; however, in the LND- group, it was only 29% (Fig. 1). The median age of the cohort was 64 years (IQR: 57–69), with a slight male predominance (male 64.2%, female 35.8%). Pure cholangiocarcinoma accounted for 57% of the cases (*n* = 102), whereas combined HCC-CCA accounted for 43% (*n* = 77). Most tumors were located peripherally (154 patients, 86%), with only 25 patients (14%) having perihilar tumors.

Marked differences between tumor types and clinical characteristics were observed between the LND + and LND- groups (Table 1). Patients who underwent lymphadenectomy were more likely to be female (56% vs. 27%, *p* < 0.001), have perihilar tumors (33% vs. 6%, *p* < 0.001), undergo open surgery (65% vs. 37%, *p* < 0.001), and have a preoperative diagnosis of cholangiocarcinoma (70% vs. 29%, *p* < 0.001).

**Table 1:** The clinical demographics of intrahepatic cholangiocarcinoma patients were compared between those who received lymph node dissection and those who did not

	Overall^1^	LND (+)^1^	LND (-)^1^	*p* value^2^
	*N* = 179	*N* = 54	*N* = 125	
Sex, F:M	64:115 (36:64)	30:24 (56:44)	34:91 (27:73)	< 0.001
Age, year	64 (57, 69)	63 (54, 69)	64 (58, 70)	0.33
BMI, kg/m2	25.2(22.4, 27.2)	23.9 (22.2, 27.2)	25.2 (22.6, 27.2)	0.28
ECOG 0-1 2–3	161 (90) 18 (10)	49 (91) 5 (9.3)	112 (90) 13 (10)	0.80
CCI	2 (0, 4)	2 (0, 3)	2 (1, 4)	0.02
ASA				0.80
1–2	94 (53)	29 (54)	65 (52)	
3–4	85 (47)	25 (46)	60 (48)	
HBV/HCV	124 (69)	33 (61)	91 (73)	0.12
Liver cirrhosis	26 (15)	2 (3.7)	24 (19)	0.007
History of HCC	17 (9.5)	2 (3.7)	15 (12)	0.08
Jaundice	10 (5.6)	6 (11)	4 (3.2)	0.07
CA19-9, U/ml	33 (16, 149)	48 (20, 158)	22 (12, 67)	0.03
Missing	100 (56)	13 (24)	87 (70)	
CEA, ng/ml	2 (1, 5)	3 (1, 5)	2 (1, 4)	0.48
Missing	116 (65)	21 (39)	95 (76)	
AFP, ng/ml	5 (3, 26)	4 (2, 10)	5 (3, 28)	0.046
Missing	34 (19)	11 (20)	23 (18)	
Tumor numbers Single Multiple	163 (91) 16 (9)	47 (87) 7 (13)	116 (93) 9 (7)	0.11
Tumor Location Perihilar Peripheral	25 (14) 154 (86)	18 (33) 36 (67)	7 (6) 118 (94)	< 0.001
MIS	98 (55)	19 (35)	79 (63)	< 0.001
Hepatectomy MH PH	59 (33) 120 (67)	32 (59) 22 (41)	27 (22) 98 (78)	< 0.001
CBD excision	16 (8.9)	14 (26)	2 (1.6)	< 0.001
OP time, min	215 (155, 313)	279 (196, 368)	197 (141, 272)	< 0.001
EBL, ml	300 (100, 500)	400 (150, 800)	200 (0, 450)	< 0.001
LOS, day	7 (6, 11)	10 (7, 15)	7 (5, 10)	< 0.001
ICU stay, day	0 (0, 3)	0 (0, 4)	0 (0, 2)	0.009
Complications				0.023
CD grade 3/4 Mortality	18 (10) 3 (1.7)	7 (13) 3 (5.6)	11 (8.8) 0	

*CCI* Charlson Comorbidity Index, *HCC* hepatocellular carcinoma, *MIS* minimally invasive surgery, *MH* major hepatectomy (tri-segmentectomy or lobectomy), *PH* partial hepatectomy, *EBL* Estimated blood loss, *LOS* Length of hospital stay, *CD* Clavien‒Dindo grade

^1^Median (Q1, Q3) or frequency (%). ^*2*^ Fisher’s exact test; Pearson’s chi-square test

*Missing tumor markers were primarily from patients with a nonpreoperative cholangiocarcinoma diagnosis

Patients selected for lymphadenectomy demonstrated significantly more aggressive tumor characteristics (Table 2). These patients had larger tumors (median 4.0 vs. 3.0 cm, *p* < 0.001), more frequent mixed growth patterns (28% vs. 6%, *p* < 0.001), higher rates of perineural invasion (30% vs. 8.8%, *p* < 0.001), and more advanced pathological stages (39% vs. 13% stage 3a/3b, *p* < 0.001). Among patients who underwent lymphadenectomy, adequate lymph node harvest (≥ 6 nodes) was achieved in only 14 patients (26%).

**Table 2:** Pathological characteristics of intrahepatic cholangiocarcinoma patients compared between those who received lymph node dissection and those who did not

	Overall^1^	LND (+)^1^	LND (-)^1^	*p* value^2^
	*N* = 179	*N* = 54	*N* = 125	
Cell type				< 0.001
CCA	102 (57)	44 (81)	58 (46)	
HCC-CCA	77 (43)	10 (19)	67 (54)	
Differentiation				0.78
Well	12 (6.7)	4 (7.4)	8 (6.4)	
Moderate	128 (71.5)	40 (74)	88 (70.4)	
Poor/UD	39 (21.8)	10 (19)	29 (23.2)	
Tumor size, cm	3.3 (2.2, 4.6)	4.0 (3.0, 5.5)	3.0 (2.0, 4.1)	< 0.001
TGP				< 0.001
Mass-forming	156 (87)	39 (72)	117 (94)	
Mixed type	23 (13)	15 (28)	8 (6)	
LVI	15 (8.4)	6 (11)	9 (7.2)	0.69
SVI	67 (37)	25 (46)	42 (34)	0.11
PNI	27 (15)	16 (30)	11 (8.8)	< 0.001
Margin status*				0.64
R0	149 (83)	43 (80)	106 (85)	
R1	15 (8.4)	6 (11)	9 (7.2)	
R2	15 (8.4)	5 (9.3)	10 (8)	
P-Stage				< 0.001
1a/1b	83 (46)	19 (35)	64 (51)	
2	59 (33)	14 (26)	45 (36)	
3a/3b	37 (21)	21 (39)	16 (13)	
Adequate LND (≧ 6 nodes)	14 (7.8)	14 (26)	0	< 0.001
P-N stage				< 0.001
pN0	41 (23)	41 (76)	0	
pN1	13 (7)	13 (24)	0	
pNx	125 (70)	0	125 (100)	

*CCA* pure cholangiocarcinoma, *HCC-CCA* combined hepato-cholangiocarcinoma, *UD* undifferentiated, *TGP* tumor growth pattern. The mixed type combines the mass-forming and peri-ductal infiltrating types. LVI, large vessel invasion. SVI, small vessel invasion. P-Stage, Pathological Staging, according to the definition of the AJCC 8th edition

^1^Median (Q1, Q3) or frequency (%)

^2^Fisher’s exact test; Pearson’s chi-square test

* The 1 mm rule defines the margin status for R0 (> 1 mm) and R1 (≤ 1 mm); an involved margin is referred to as R2

## Tumor markers

Preoperative tumor markers were inconsistently obtained, including Carbohydrate antigen 19 − 9 (CA19-9), Carcinoembryonic Antigen (CEA), and Alpha-fetoprotein (AFP) (Table 1). CA19-9 was available in only 44% of patients overall, with markedly higher missing rates in the LND- group (70%) compared to the LND + group (24%); median values were 48 (IQR: 20–158) and 22 (IQR: 12–67) U/ml, respectively. AFP was available in 80% of patients, with median values of 4 ng/mL (IQR: 2–10) in the LND + group and 5 ng/mL (IQR: 3–28) in the LND- group (*p* = 0.046). Neither marker demonstrated prognostic significance for overall survival nor influenced lymphadenectomy decisions in logistic regression analysis. The high rate of missing tumor markers, particularly in the LND- group, further supports the diagnostic uncertainty inherent in this patient population.

## Neoadjuvant and adjuvant therapy

The administration of neoadjuvant chemotherapy in intrahepatic cholangiocarcinoma was limited to 13 patients (7.3%), primarily within the CCA group, who received Gemcitabine plus Cisplatin or Gemcitabine/Cisplatin plus Durvalumab (ESM_Table_1). In the HCC-CCA group, three patients received neoadjuvant treatment for HCC, including Atezolizumab plus Bevacizumab and Hepatic Arterial Infusion Chemotherapy using Cisplatin plus Doxorubicin. Meanwhile, one case in CCA also received Pembrolizumab plus Lenvatinib, highlighting the limitations of preoperative diagnosis.

Only 38 patients (21.2%) received adjuvant therapy, including twenty-five in the LND (+) group and thirteen in the LND (-) group. The conduction of adjuvant treatment was significantly higher in the LND (+) than LND (-) patients, regardless of the CCA (45% vs. 10%) or HCC-CCA group (50% vs. 10%). The most used regimen were Gemcitabine plus Cisplatin-based treatment (36.8%) and S-1 monotherapy (39.5%).

### Factors associated with lymphadenectomy decisions

Logistic regression in univariable analysis revealed that patients with a tumor size larger than 3 cm (OR: 2.66, 95% CI 1.36–5.36, *p* = 0.003), preoperative CCA diagnosis (OR: 5.88, 95% CI 2.96–12.05, *p* < 0.001), and major hepatectomy, such as trisectionectomy or lobectomy (OR: 5.29, 95% CI 2.67–10.64, *p* < 0.001), were associated with the decision to perform lymphadenectomy. Moreover, liver cirrhosis (OR: 0.16, 95% CI 0.03–0.58, *p* = 0.016), a peripherally located tumor (OR: 0.12, 95% CI 0.04–0.30, *p* < 0.001), and minimally invasive surgery (OR: 0.32, 95% CI 0.16–0.61, *p* < 0.001) were associated with the decision “not” to perform lymphadenectomy (Table 3).

**Table 3:** Logistic regression model to assess the factors associated with the decision to undergo lymph node dissection

	Lymph Node Dissection (+)	Lymph Node Dissection (-)	Univariable		Multivariable	
	*N* = 54	*N* = 125	OR (95% CI)	*p* value	OR (95% CI)	*p* value
Liver cirrhosis	2 (3.7)	24 (19.2)	0.16 (0.03–0.58)	0.016	0.28 (0.04–1.17)	0.12
Tumor > 3 cm	38 (70.4)	59 (47.2)	2.66 (1.36–5.36)	0.003	1.36 (0.60–3.05)	0.68
Location, peripheral type	36 (66.7)	118 (94.4)	0.12 (0.04–0.30)	< 0.001	0.41 (0.13–1.28)	0.13
Preoperative diagnosis of BTC	38 (70.4)	36 (28.8)	5.88 (2.96–12.05)	< 0.001	3.33 (1.54–7.34)	0.002
Minimally invasive surgery	19 (35.2)	79 (63.2)	0.32 (0.16–0.61)	< 0.001	0.50 (0.23–1.09)	0.08
Major hepatectomy	32 (59.3)	27 (21.6)	5.29 (2.67–10.64)	< 0.001	1.73 (0.70–4.22)	0.23

According to the multivariate analysis, patients with a preoperative diagnosis of cholangiocarcinoma were more than three times more likely to undergo lymphadenectomy (OR 3.33, 95% CI 1.54–7.34; *p* = 0.002). Other factors that were significant in the univariable analysis, including liver cirrhosis, tumor size, tumor location, and surgical approach, lost significance after adjustment for preoperative diagnosis.

### Survival outcomes

**Fig. 2:**
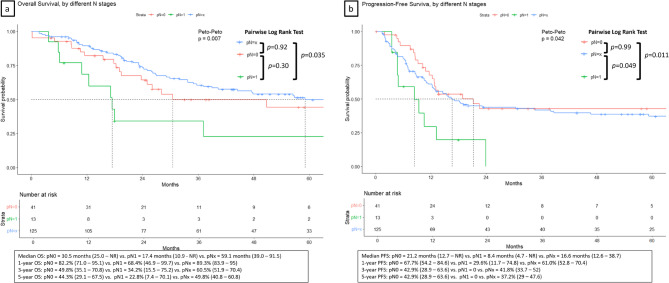
Kaplan-Meier survival curves showing (**A**) overall survival and (**B**) progression-free survival by pathological nodal status (pN0, pN1, and pNx) in patients with intrahepatic cholangiocarcinoma

With a median follow-up of 27 months, 87 patients (49%) died during the study period. The median OS varied significantly by nodal status: 30.5 months (95% CI 25.0–NR), 17.4 months (95% CI 10.9–NR), and 59.1 months (95% CI 39.0–91.5) (Peto-Peto test *p* = 0.007) for pN0, pN1, and pNx, respectively; the median PFS were 21.2 months (95% CI 12.7–NR), 8.4 months (95% CI 4.7–NR), and 16.6 months (95% CI 12.6–38.7) (Fig. 2A-B). Pairwise Log-rank test with Bonferroni correction revealed a significant difference for OS between pNx vs. pN1 (*p* = 0.035), but no difference comparing pN0 vs. pN1 and pN0 vs. pNx; the comparison for PFS was significant between pN0 vs. pN1 (*p* = 0.011) and pNx vs. pN1 (*p* = 0.049). The five-year OS and PFS rates were 44.3% (95% CI 29.1–67.5) vs. 22.8% (95% CI 7.4–70.1) vs. 49.8% (95% CI 40.8–60.8), and 42.9% (95% CI 28.9–63.6) vs. 0% vs. 37% (95% CI 29–47.6) for pN0 vs. pN1 vs. pNx, respectively.

Univariable Cox regression analysis identified several independent prognostic factors for overall survival (Table 4). There was no significant difference in survival between patients in the pNx vs. pN0 group (HR 0.78, 95% CI 0.46–1.30; *p* = 0.34). Independent predictors of worse OS in the multivariable analysis included advanced age ≥ 65 years (HR 1.71, 95% CI 1.10–2.64, *p* = 0.016), advanced pathological stage (stage 2 vs. stage 1: HR 1.89, 95% CI 1.10–3.24, *p* = 0.021; stage 3 vs. stage 1: HR 2.89, 95% CI 1.52–5.51, *p* = 0.001), and pure cholangiocarcinoma histology compared with combined HCC-CCA (HR 1.68, 95% CI 1.03–2.75, *p* = 0.040).

**Table 4:** Cox regression models of risk factors for overall survival

	Mortality*N* = 87	Survival*N* = 92	Univariable		Multivariable	
			HR (95% CI)	*p* value	HR (95% CI)	*p* value
Age ≥ 65	49 (56.3)	36 (39.1)	1.78 (1.16–2.73)	0.008	1.71 (1.10–2.64)	0.016
Sex, Male	51 (58.6)	64 (69.6)	0.70 (0.46–1.08)	0.10		
pN status						
pN0	19 (21.8)	22 (23.9)	Ref.	Ref.		
pN1	9 (10.3)	4 (4.3)	1.89 (0.85–4.21)	0.12		
pNx	59 (67.8)	66 (71.7)	0.78 (0.46–1.30)	0.34		
P-Staging						
Stage1	28 (32.2)	55 (59.8)	Ref.	Ref.	Ref.	Ref.
Stage 2	31 (35.6)	28 (30.4)	1.97 (1.18–3.29)	0.009	1.89 (1.10–3.24)	0.021
Stage 3	28 (32.2)	9 (9.8)	3.32 (1.97–5.62)	< 0.001	2.89 (1.52–5.51)	0.001
Tumor > 3 cm	57 (65.5)	40 (43.5	1.75 (1.12–2.72)	0.013	1.36 (0.84–2.20)	0.22
Location						
Peripheral	68 (78.2)	86 (93.5)	Ref.	Ref.	Ref.	Ref.
Perihilar	19 (21.8)	6 (6.5)	2.08 (1.25–3.47)	0.005	1.05 (0.59–1.86)	0.87
Cell type						
HCC-CCA	29 (33.3)	48 (52.2)	Ref.	Ref.	Ref.	Ref.
CCA	58 (66.7)	44 (47.8)	1.92 (1.23-3.00)	0.004	1.68 (1.03–2.75)	0.040
Margin						
R0	67 (77)	82 (89.1)	Ref.	Ref.	Ref.	Ref.
R1/2 vs. R0	20 (23)	10 (10.9)	1.74 (1.05–2.87)	0.031	1.21 (0.71–2.07)	0.49
Adjuvant	19 (21.8)	19 (20.7)	1.67 (1.00-2.80)	0.049	0.85 (0.46–1.58)	0.61

*pN0,* lymphadenectomy performed, negative nodes; *pN1 *lymphadenectomy performed, positive nodes, pNx: no lymphadenectomy performed

The group of pNx demonstrated superior overall survival and without increased hazard for PFS in univariable analysis (pNx vs. pN0: HR 1.21, 95% CI 0.74–1.99) (Table 5). However, after adjusting for tumor stage, tumor size, location, histological type, margin status, and adjuvant therapy or not, pNx became an independent risk factor with increased hazard to 2.12 (95% CI 1.20–3.77, *p* = 0.012) compared to the group of pN0. Other independent predictors of worse PFS in the multivariable analysis included advanced pathological stage (stage 2 vs. stage 1: HR 2.56, 95% CI 1.60–4.11, *p* < 0.001; stage 3 vs. stage 1: HR 3.46, 95% CI 1.86–6.44, *p* < 0.001), and pure cholangiocarcinoma histology compared with combined HCC-CCA (HR 1.59, 95% CI 1.02–2.48, *p* = 0.042).

**Table 5:** Cox regression models of risk factors for progression-free survival

	Recurrence *N* = 104	No Recurrence *N* = 75	Univariable		Multivariable	
			HR (95% CI)	*p* value	HR (95% CI)	*p* value
Age ≥ 65	52 (50)	33 (44)	1.23 (0.84–1.81)	0.30		
Sex, Male	67 (64.4)	48 (64)	0.90 (0.61–1.35)	0.62		
pN status						
pN0	20 (19.2)	21 (28)	Ref.	Ref.	Ref.	Ref.
pN1	10 (9.6)	3 (4)	2.78 (1.30–5.98)	0.009	1.22 (0.51–2.94)	0.65
pNx	74 (71.2)	51 (68)	1.21 (0.74–1.99)	0.45	2.12 (1.20–3.77)	0.012
P-Staging						
Stage1	33 (31.7)	50 (66.7)	Ref.	Ref.	Ref.	Ref.
Stage 2	42 (40.4)	17 (22.7)	2.52 (1.59–3.99)	< 0.001	2.56 (1.60–4.11)	< 0.001
Stage 3	29 (27.9)	8 (10.6)	3.89 (2.34–6.47)	< 0.001	3.46 (1.86–6.44)	< 0.001
Tumor > 3 cm	64 (61.5)	33 (44)	1.73 (1.16–2.57)	0.007	1.44 (0.93–2.22)	0.102
Location						
Peripheral	88 (84.6)	66 (88)	Ref.	Ref.	Ref.	Ref.
Perihilar	16 (15.4)	9 (12)	2.08 (1.25–3.47)	0.005	1.04 (0.56–1.90)	0.91
Cell type						
HCC-CCA	39 (37.5)	38 (50.7)	Ref.	Ref.	Ref.	Ref.
CCA	65 (62.5)	37 (49.3)	1.53 (1.03–2.28)	0.035	1.59 (1.02–2.48)	0.042
Margin						
R0	80 (76.9)	69 (92)	Ref.	Ref.	Ref.	Ref.
R1/2 vs. R0	24 (23.1)	6 (8)	1.77 (1.12–2.81)	0.014	1.42 (0.86–2.34)	0.17
Adjuvant	26 (25)	12 (16)	1.85 (1.18–2.92)	0.008	1.45 (0.84–2.52)	0.19

pN0: lymphadenectomy performed, negative nodes; pN1: lymphadenectomy performed, positive nodes; pNx: no lymphadenectomy performed

A total of 104 patients (58.1%) suffered from recurrence after the operation, including 64 cases (61.5%) with liver recurrence, followed by 22 cases (21.2%) with para-aortic or peri-portal lymph node recurrence, and eight cases (7.7%) with lung or pleural recurrence. The highest rate of recurrence was noted in the pN1 group (77%), followed by the pNx (59%) and the pN0 (49%). The rate of liver recurrence was 38% vs. 40% vs. 22%, and the rate of lymph node recurrence was 23% vs. 13% vs. 7.3% in pN1 vs. pNx vs. pN0, respectively. Among the subset of CCA, the lymph node recurrence rate was not different comparing the patients with (14%) and without (17%) lymphadenectomy. Among the subset of HCC-CCA, there’s no lymph node recurrence in the lymphadenectomy group; however, 9% lymph node recurrence was detected.

### Subgroup analysis

**Fig. 3:**
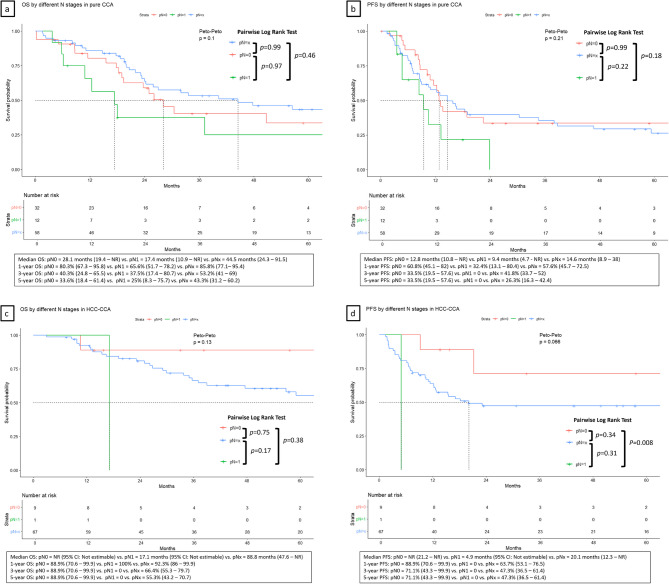
Composite figure showing overall survival by lymphadenectomy status stratified by tumor type: (**A**-**B**) pure CCA and (**C**-**D**) HCC-CCA subgroups

Subgroup survival analysis was performed for CCA (Fig. 3A-B) and HCC-CCA (Fig. 3C-D). In the pure CCA subgroup (*n* = 102), patients who underwent lymphadenectomy demonstrated similar survival patterns to the overall cohort, with median OS of 28.1 months for pN0, 17.4 months for pN1, and 44.5 months for pNx patients. In the HCC-CCA subgroup (*n* = 77), the median OS was not reached for pN0, 17.1 months for pN1, and 88.8 months for pNx patients. However, these differences did not reach statistical significance due to smaller sample sizes in each group.

Cox regression analysis was performed in CCA and HCC-CCA separately for OS and PFS. The group of pNx and pN1 vs. pN0 showed no significant increase of hazard for OS in both subgroups of pure CCA (ESM_Table_2) and combined HCC-CCA (ESM_Table_3). Because of the limited case numbers in each group, the increase hazard for pN1 cannot be demonstrated in this subgroup analysis, leaving the only independent risk factor for OS as pathological stage 3 (HR: 2.43 (95% CI 1.18-5.00) in CCA; 3.64 (95% CI 1.07–12.46) in HCC-CCA) versus stage 1. The Cox regression analysis result for PFS revealed a similar pattern in both subgroups (ESM_Table_4 for CCA and ESM_Table_5 for HCC-CCA). In the multivariable analysis, significant risk factors were advanced pathological stages in both subsets, age older than 65 years in the CCA group, and positive surgical margin in the HCC-CCA group. Compared to the Cox regression for PFS in the entire cohort, pNx showed a trend of increased hazard in both subsets without statistical significance.

## Discussion

This study offers valuable insights into real-world lymph node management practices for intrahepatic cholangiocarcinoma, challenging the uniform application of routine lymphadenectomy recommendations. Our primary finding that selective lymphadenectomy does not compromise survival outcomes has significant implications for clinical practice and guideline development.

The most striking finding was that patients who did not undergo lymphadenectomy (pNx) not only had similar survival outcomes to those who underwent lymphadenectomy with negative nodes (pN0) but also achieved superior median OS (59.1 vs. 30.5 months), with no significant difference in adjusted overall survival analysis (HR 0.78, *p* = 0.34). On the other hand, these findings require careful interpretation. The pNx group had higher hazard for PFS (HR 2.12, *p* = 0.012) compared to pN0 group after adjusting with the pathological stages and CCA vs. HCC-CCA. The biologically counterintuitive finding reflects the real-world surgical decision-making where surgeons reserved lymphadenectomy for patients with higher clinical suspicion of cholangiocarcinoma (OR 3.33, *p* = 0.002). The pNx group contained a higher proportion of HCC-CCA cases (54% vs. 19% in LND + group), which demonstrated inherently better prognosis in our analysis. Additionally, patients in the pNx group had smaller tumors (median 3.0 vs. 4.0 cm), less advanced pathological stages, and fewer high-risk features, suggesting they represented a lower-risk population that may not have required the more extensive surgical evaluation.

Three key lines of evidence support selective rather than universal lymphadenectomy approaches for iCCA. First, individual cohort studies demonstrate heterogeneous survival outcomes, with several high-quality propensity score-matched analyses showing no significant survival differences between lymphadenectomy and non-lymphadenectomy groups (ESM_Table_6) [14, 24–28]. Kim et al. found no significant survival difference after propensity score matching [29], while Yoh et al. demonstrated the prognostic value of lymphadenectomy specifically in node-negative patients [30]. The variability in these findings may reflect differences in patient selection, surgical techniques, and tumor characteristics across institutions [24, 31].

Second, location-specific analyses reveal differential benefits based on tumor characteristics. Umeda et al. demonstrated that lymph node dissection had a prognostic impact for hilar-type iCCA but lacked therapeutic benefit for peripheral-type tumors [25], consistent with our subgroup analysis showing location-dependent outcomes. Additionally, recent efforts to develop prediction models for lymph node metastasis may help identify patients who would benefit most from lymphadenectomy [32, 33]. For perihilar tumors, the high LND rate (72%) reflects appropriate surgical practice, as these tumors have a greater propensity for lymphatic spread and often require more extensive resection, including portal lymphadenectomy. However, even in this high-risk subgroup, we found no survival detriment for the few patients (28%) who did not undergo LND, though the small sample size (*n* = 25) limits statistical power.

Third, systematic reviews and meta-analyses consistently fail to demonstrate clear survival benefits from routine lymphadenectomy (ESM_Table_7) [34–37]. Atif et al. conclude that LND may aid in staging, prognosticating, and determining further management of resected ICCA, but not improving OS and DFS [38]. The most recent meta-analysis by Yeow et al. found no significant overall survival benefit (HR 0.78, *p* = 0.11) [36], while other systematic reviews reached similar conclusions [37]. The concept that quality rather than universality may be more important is supported by studies examining adequate lymphadenectomy thresholds, with survival benefits observed only in patients receiving sufficient lymphadenectomy (≥ 6 nodes) [26, 27, 39].

The increasing adoption of minimally invasive surgical approaches adds complexity to lymphadenectomy decisions due to technical challenges and potential complications. The feasibility and safety of laparoscopic lymphadenectomy have been demonstrated, but the procedure remains technically demanding and may not be universally applicable [40].

Combined HCC-CCA presents unique management challenges due to its mixed cellular characteristics and the frequent occurrence of preoperative misdiagnosis. The superior survival of combined HCC-CCA compared to pure cholangiocarcinoma (HR 1.68, *p* = 0.040) in our study represents a significant finding. This observation contrasts with traditional assumptions about mixed tumors and may reflect different biological behaviors [9, 11]. Recent molecular studies have revealed that combined HCC-CCA tumors derive from liver progenitor cells and depend on different pathways than pure cholangiocarcinoma [41], potentially explaining their distinct clinical behavior. Despite these biological differences and characteristic patterns of lymphatic spread between tumor types, our findings suggest that selective lymphadenectomy approaches are appropriate for both entities, supporting a unified clinical strategy based on preoperative suspicion rather than histological subtype. Long-term survival data for combined HCC-CCA have shown favorable outcomes in some series, particularly when complete resection is achieved [11].

The impact of various prognostic factors beyond lymph node status, including tumor size, location, and resection margins, has been extensively studied [42, 43]. Primary tumor characteristics often outweigh nodal status in determining long-term outcomes, particularly in the era of improved perioperative care and adjuvant therapy options.

Our study highlights the ongoing challenge of achieving adequate lymphadenectomy. Only 26% of patients who underwent lymphadenectomy had ≥ 6 lymph nodes retrieved, consistent with other studies showing low rates in real-world practice and raising questions about the practical feasibility of routine comprehensive lymph node dissection.

The implications for clinical practice are significant. Rather than mandating routine lymphadenectomy for all iCCA patients, our results support individualized decision-making based on clinical factors, particularly preoperative diagnostic certainty. This approach preserves oncological outcomes while potentially reducing surgical morbidity and operative complexity.

From a guideline perspective, our findings suggest that current recommendations for routine lymphadenectomy may need refinement. A more nuanced approach that considers tumor characteristics, location, and preoperative diagnostic confidence may be more clinically relevant than universal recommendations. The evolving understanding of cholangiocarcinoma biology and the development of new therapeutic options, including targeted therapies and immunotherapy, may further influence the role of surgical staging in treatment planning [8, 20–22]. While our findings challenge routine lymphadenectomy from a survival perspective, it is important to acknowledge that LND serves critical roles in staging. However, these staging benefits must be weighed against surgical complexity, potential morbidity, and the reality that adequate lymphadenectomy (≥ 6 nodes) is achieved in only 26% of cases in real-world practice. Our results suggest that the staging value of LND may be most appropriately reserved for cases where this information will meaningfully impact treatment decisions.

Geographic and institutional variations in iCCA presentation and management approaches, as reflected in the recent development of Pan-Asian adapted guidelines [14], further support the need for individualized approaches. The integration of molecular profiling and precision medicine approaches may eventually supersede traditional anatomical staging in determining treatment strategies.

Several limitations must be acknowledged. As a retrospective, single-center study, our findings may not be generalizable to all practice settings. Selection bias, while central to our analysis, cannot be completely controlled for in observational studies. Another significant limitation is the temporal disconnect between intraoperative decision-making (based on clinical suspicion) and our analysis based on final pathological diagnosis. Surgeons made lymphadenectomy decisions without knowing the final histological subtype, yet our analysis stratifies outcomes by pure CCA versus HCC-CCA. This creates an inherent bias where the most convincing clinical presentations of cholangiocarcinoma were more likely to undergo lymphadenectomy, regardless of final pathology. The relatively short median follow-up of 27 months may not capture long-term survival differences and the late recurrences in cholangiocarcinoma. Yet, in our analysis, most of the patients developed recurrence within two years after the operation. Additionally, the inclusion of both pure cholangiocarcinoma and combined HCC-CCA, while reflecting real-world practice, introduces tumor heterogeneity that may mask subtype-specific effects in this moderately-sized cohort.

## Conclusions

This study demonstrated that, compared with routine lymphadenectomy with negative nodes, selective lymph node dissection for intrahepatic cholangiocarcinoma does not compromise survival outcomes. These findings support individualized surgical approaches on the basis of clinical judgment rather than universal lymphadenectomy protocols. Future guidelines should incorporate more nuanced recommendations that acknowledge iCCA heterogeneity and support risk-stratified decision-making in lymph node management.

## Supplementary Information

Supplementary Material 1.

## Data Availability

The datasets used in the study are available from the corresponding author upon reasonable request.
