# Supplementary material for: Selective lymph node dissection in intrahepatic cholangiocarcinoma and combined hepatocellular cholangiocarcinoma may not impair oncological outcomes: a single-center retrospective cohort study
Source: World J Surg Oncol. 2025 Oct 21;23:387. doi: 10.1186/s12957-025-04034-3 (PMC12539170; doi:10.1186/s12957-025-04034-3)

**Selective lymph node dissection in intrahepatic cholangiocarcinoma and combined hepatocellular cholangiocarcinoma may not impair oncological outcomes: A single-center retrospective cohort study**

**World Journal of Surgical Oncology**

**Authors:**

Wei-Hsun Lu, M.D.^1, 2*^, Ting-Kai Liao, M.D.^1,2^, Che-Min Su, M.D. ^1^, Tsung-Han Yang, M.D. ^1^, Tsung-Ching Chou^1^, Ping-Jui Su, M.D.^1^, Chih-Jung Wang, M.D.^1, 2^, Ying Jui Chao, M.D., Ph.D. ^1, 2^, Yih-Jyh Lin, M.D.^1^, Yan-Shen Shan, M.D., Ph.D.^1, 2^

1. Department of Surgery, National Cheng Kung University Hospital, College of Medicine, National Cheng Kung University, Tainan, Taiwan
2. Institute of Clinical Medicine, College of Medicine, National Cheng Kung University, Tainan, Taiwan

**Address correspondence and reprint requests to:**

Ting-Kai Liao M.D.

Department of Surgery, National Cheng Kung University Hospital; Institute of Clinical Medicine, College of Medicine, National Cheng Kung University, Tainan, Taiwan. 138, Sheng-Li Road, Tainan 70428, Taiwan

TEL: 886-6-235 3535 #5181, Fax: 886 6 276 6676

Email: [n049550@mail.hosp.ncku.edu.tw](mailto:n049550@mail.hosp.ncku.edu.tw)

ORCID: 0000-0003-4742-8066

**ESM_Table_1.** The conduction of neoadjuvant and adjuvant therapy in the group of CCA and HCC-CCA, comparing the subgroups with and without lymph node dissection.

|  | **CCA** | | **HCC-CCA** | |
| --- | --- | --- | --- | --- |
|  | **LND (+)*^1^*** | **LND (-)*^1^*** | **LND (+)*^1^*** | **LND (-)*^1^*** |
|  | **N=44** | **N=58** | **N=10** | **N=67** |
| NAC | 9 (20) | 1 (1.7) | 0 (0) | 3 (4.5) |
| NAC regimen |  |  |  |  |
| Gem+Cis | 3 |  |  |  |
| Gem+S-1 | 1 |  |  |  |
| Gem+Cis+Durvalumab | 3 |  |  |  |
| Gem+S1+Nivolumab | 1 | 1 |  |  |
| Pembrolizumab+Lenvatinib | 1 |  |  |  |
| Atezolizumab+Bevacizumab |  |  |  | 1 |
| HAIC |  |  |  | 2 |
| Adjuvant therapy | 20 (45) | 6 (10) | 5 (50) | 7 (10) |
| Gem+Cis | 7 (35) | 3 (50) |  | 2 (35.7) |
| S-1 | 7 (35) | 2 (33.3) | 4 (40) | 2 (35.7) |
| Capecitabine | 0 | 1 (16.7) |  |  |
| Cisplatin + HDFL | 3 (15) |  |  |  |
| Sorafenib |  |  | 1 (10) | 1 (14.3) |
| Gem+Cis+Durvalumab | 2 (10) |  |  |  |
| Gem+S1+Nivolumab | 1 (5) |  |  |  |
| Atezolizumab+Bevacizumab |  |  |  | 1 (14.3) |
| HAIC |  |  |  | 1 (14.3) |

Gem, Gemcitabine. Cis, Cisplatin. HAIC, Hepatic Arterial Infusion Chemotherapy; the regimen of HAIC is Cisplatin plus Doxorubicin.

**ESM_Table_2.** Cox regression models of risk factors for overall survival in CCA group.

|  | Mortality  N=58 | Survival  N=44 | Univariable | | Multivariable | |
| --- | --- | --- | --- | --- | --- | --- |
|  |  |  | HR (95% CI) | *p* value | HR (95% CI) | *p* value |
| Age ≥ 65 | 33 (56.9) | 16 (36.4) | 1.66 (0.98-2.79) | 0.06 | 1.47 (0.85-2.54) | 0.17 |
| Sex, Male | 32 (55.2) | 27 (61.4) | 0.74 (0.44-1.25) | 0.26 |  |  |
| pN status  pN0  pN1  pNx | 18 (31)  8 (13.8)  32 (55.2) | 14 (31.8)  4 (9.1)  26 (59.1) | Ref.  1.43 (0.62-3.32)  0.79 (0.44-1.42) | Ref.  0.41  0.44 |  |  |
| P-Staging  Stage1  Stage 2  Stage 3 | 20 (34.5) 16 (27.6)  22 (37.9) | 26 (59.1)  13 (29.5)  5 (11.4) | Ref.  1.71 (0.88-3.31)  3.18 (1.73-5.86) | Ref.  0.11  <0.001 | Ref.  1.56 (0.77-3.18)  2.43 (1.18-5.00) | Ref.  0.22  0.016 |
| Tumor>3 cm | 40 (69) | 18 (40.9) | 1.79 (1.02-3.12) | 0.042 | 1.37 (0.75-2.50) | 0.30 |
| Location  Peripheral  Perihilar | 41 (70.7)  17 (29.3) | 40 (90.9)  4 (9.1) | Ref.  1.91 (1.08-3.37) | Ref.  0.026 | Ref.  0.88 (0.47-1.65) | Ref.  0.68 |
| Margin  R0  R1/2 vs R0 | 42 (72.4)  16 (27.6) | 36 (81.8)  8 (18.2) | Ref.  1.17 (0.66-2.09) | Ref.  0.59 |  |  |
| Adjuvant | 14 (24.1) | 12 (27.3) | 1.27 (0.69-2.32) | 0.44 |  |  |

Variables with p<0.10 in univariable analysis were included in the multivariable analysis.

**ESM_Table_3.** Cox regression models of risk factors for overall survival in HCC-CCA group.

|  | Mortality  N=29 | Survival  N=48 | Univariable | | Multivariable | |
| --- | --- | --- | --- | --- | --- | --- |
|  |  |  | HR (95% CI) | *p* value | HR (95% CI) | *p* value |
| Age ≥ 65 | 16 (55.2) | 20 (41.7) | 1.89 (0.90-3.99) | 0.09 | 1.98 (0.77-5.12) | 0.16 |
| Sex, Male | 19 (65.5) | 37 (77.1) | 0.76 (0.36-1.64) | 0.49 |  |  |
| pN status  pN0  pN1  pNx | 1 (3.4)  1 (3.4)  27 (93.1) | 8 (16.7)  0 (0)  40 (83.3) | Ref.  16.67 (1.01-275.10)  3.02 (0.41-22.27) | Ref.  0.049  0.28 | Ref.  10.94 (0.59-204.08)  2.36 (0.30-18.57) | Ref.  0.11  0.41 |
| P-Staging  Stage1  Stage 2  Stage 3 | 8 (27.6) 15 (51.7)  6 (20.7) | 29 (60.4)  15 (31.3)  4 (8.3) | Ref.  2.85 (1.21-6.75)  2.87 (0.99-8.31) | Ref.  0.017  0.05 | Ref.  2.22 (0.88-5.63)  3.64 (1.07-12.46) | Ref.  0.09  0.039 |
| Tumor>3 cm | 17 (58.6) | 22 (45.8) | 1.53 (0.73-3.22) | 0.26 |  |  |
| Location  Peripheral  Perihilar | 27 (93.1)  2 (6.9) | 46 (95.8)  2 (4.2) | Ref.  1.33 (0.32-5.61) | Ref.  0.70 |  |  |
| Margin  R0  R1/2 vs R0 | 25 (86.2)  4 (13.8) | 46 (95.8)  2 (4.2) | Ref.  4.05 (1.36-12.08) | Ref.  0.012 | Ref.  2.68 (0.83-8.69) | Ref.  0.10 |
| Adjuvant | 4 (13.8) | 2 (4.2) |  |  |  |  |

Variables with p<0.10 in univariable analysis were included in the multivariable analysis.

**ESM_Table_4.** Cox regression models of risk factors for progression-free survival in CCA group.

|  | Recurrence  N=65 | No Recurrence  N=37 | Univariable | | Multivariable | |
| --- | --- | --- | --- | --- | --- | --- |
|  |  |  | HR (95% CI) | *p* value | HR (95% CI) | *p* value |
| Age ≥ 65 | 35 (53.8) | 14 (37.8) | 1.60 (0.98-2.61) | 0.06 | 1.63 (0.98-2.72) | 0.06 |
| Sex, Male | 38 (29.2) | 21 (100) | 0.75 (0.46-1.23) | 0.26 |  |  |
| pN status  pN0  pN1  pNx | 18 (3.4)  9 (3.4)  38 (93.1) | 8 (16.7)  0 (0)  40 (83.3) | Ref.  2.15 (0.96-4.82)  1.09 (0.62-1.91) | Ref.  0.06  0.77 | Ref.  0.99 (0.40-2.48)  1.45 (0.82-2.57) | Ref.  0.98  0.21 |
| P-Staging  Stage1  Stage 2  Stage 3 | 22 (33.8) 22 (33.8)  21 (32.3) | 24 (64.9)  7 (18.9)  6 (16.2) | Ref.  2.64 (1.45-4.80)  4.10 (2.19-7.68) | Ref.  0.001  <0.001 | Ref.  3.02 (1.64-5.56)  4.59 (2.18-9.66) | Ref.  <0.001  <0.001 |
| Tumor>3 cm | 42 (64.6) | 16 (43.2) | 2.00 (1.20-3.02) | 0.10 |  |  |
| Location  Peripheral  Perihilar | 51 (78.5)  14 (21.5) | 30 (81.1)  7 (18.9) | Ref.  1.66 (0.92-5.61) | Ref.  0.70 |  |  |
| Margin  R0  R1/2 vs R0 | 46 (70.8)  19 (29.2) | 32 (86.5)  5 (13.5) | Ref.  1.23 (0.72-2.10) | Ref.  0.45 |  |  |

Variables with p<0.10 in univariable analysis were included in the multivariable analysis.

**ESM_Table_5.** Cox regression models of risk factors for progression-free survival in HCC-CCA group.

|  | Mortality  N=39 | Survival  N=38 | Univariable | | Multivariable | |
| --- | --- | --- | --- | --- | --- | --- |
|  |  |  | HR (95% CI) | *p* value | HR (95% CI) | *p* value |
| Age ≥ 65 | 17 (43.6) | 19 (50) | 0.86 (0.46-1.63) | 0.65 |  |  |
| Sex, Male | 29 (74.4) | 27 (71.1) | 1.29 (0.63-2.64) | 0.50 |  |  |
| pN status  pN0  pN1  pNx | 2 (5.1)  1 (2.6)  36 (92.3) | 7 (18.4)  0 (0)  31 (81.6) | Ref.  14.63 (1.27-168.74)  3.01 (0.72-12.49) | Ref.  0.032  0.13 | Ref.  9.24 (0.73-116.51)  3.20 (0.76-13.43) | Ref.  0.09  0.11 |
| P-Staging  Stage1  Stage 2  Stage 3 | 11 (28.2) 20 (51.3)  8 (20.5) | 26 (68.4)  10 (26.3)  2 (5.3) | Ref.  2.77 (1.32-5.80)  3.91 (1.56-9.79) | Ref.  0.007  0.004 | Ref.  2.66 (1.25-5.66)  4.13 (1.58-10.8) | Ref.  0.011  0.004 |
| Tumor>3 cm | 22 (56.4) | 17 (44.7) | 1.43 (0.76-2.69) | 0.27 |  |  |
| Location  Peripheral  Perihilar | 37 (94.9)  2 (5.1) | 36 (94.7)  2 (5.3) | Ref.  1.09 (0.26-4.52) | Ref.  0.91 |  |  |
| Margin  R0  R1/2 vs R0 | 34 (87.2)  5 (12.8) | 37 (97.4)  1 (2.6) | Ref.  4.70 (1.73-12.78) | Ref.  0.002 | Ref.  4.01 (1.42-11.38) | Ref.  0.009 |

Variables with p<0.10 in univariable analysis were included in the multivariable analysis.

**ESM_Table 6.** Comparison of survival outcomes across individual cohort studies of intrahepatic cholangiocarcinoma patients over the past five years.

| **Study** | **Year** | **N** | **Study Type** | **Groups Compared** | **Median OS (months)** | **3-year OS (%)** | **5-year OS (%)** | **Conclusion** |
| --- | --- | --- | --- | --- | --- | --- | --- | --- |
| **Current Study** | 2025 | 179 | Single-center cohort | pN0 vs pN1 vs pNx | 30.5 vs 17.4 vs **59.1** | 49.8 vs 34.2 vs **60.5** | 44.3 vs 22.8 vs **49.8** | HR 0.78 (0.46-1.30), p=0.335 |
| **Hu, et al. [42]** | 2021 | 232 | Multicenter cohort | LND+ vs LND- | 21 vs 61 | NR | 25.7 vs 50.6 | Paradoxically better survival without LND |
| **Kim, et al. [36]** | 2022 | 87 | Single-center cohort | N0 vs N+ <4 vs N+ ≥4 | 69.0 vs 28.0 vs 11.0 | NR | NR | Number of positive nodes matters |
| **Umeda, et al. [28]** | 2022 | 310 | Multicenter cohort | LND+ vs LND- | 57.2 vs NR | 48.0 vs 55.4 | 37.5 vs 44.6 | Nonsignificant after IPTW |
| **Chen, et al. [14]** | 2023 | 891 | Multicenter cohort | Adequate LND vs Others | NR | NR | ~40-55% vs ~25%* | Benefit only with ≥6 nodes |
| **Sposito, et al. [35]** | 2023 | 891 | Multicenter cohort | Adequate LND vs Others | NR | NR | ~40-55% vs ~25%* | Benefit only with ≥6 nodes |
| **Yoshino, et al. [26]** | 2025 | 142 | Single-center PSM | LND+ vs LND-(peripheral MF type) | NR | NR | NR | No significant difference after PSM |

**Abbreviations:** OS = overall survival; LND = lymph node dissection; PSM = propensity score matching; IPTW = inverse probability treatment weighting; NLND = no lymph node dissection; MF = mass-forming; NR = not reported; HR = hazard ratio; CI = confidence interval. *****Approximate values estimated from survival curves

**ESM_Table_7.** Comparison of survival outcomes across major intrahepatic cholangiocarcinoma patients in the meta-analysis.

| **Study** | **Year** | **N** | **Study Included** | **Groups** | **Primary Outcome** | HR (95% CI) | p value | Key Finding |
| --- | --- | --- | --- | --- | --- | --- | --- | --- |
| **Zhou, et al. [34]** | 2019 | 1,377 | 13 studies (2000-2018) | LND+ vs LND- | Overall Survival | 1.13 (0.94-1.36) | 0.20 | First comprehensive meta-analysis |
| **Li, et al. [31]** | 2022 | 1,790 | 17 studies (2009-2021) | LND+ vs LND- | 1-, 3-, 5-year survival | NR | 0.73 | Multi-timepoint analysis |
| **Atif, et al. [43]** | 2023 | 11,413 | 15 studies (2011-2022) | LND+ vs LND- | Overall Survival | 0.90 (0.77-1.06) | 0.22 | LND may aid in staging, prognosticating, and determining further management of resected ICCA, but does not improve OS and DFS. |
| **Yeow, et al. [33]** | 2024 | 3,776 | 8 studies (2019-2023), Propensity score-based studies only | LND+ vs LND- | Overall Survival | 0.78 (0.57-1.06) | 0.11 | No significant survival benefit |

**Abbreviations:** OS = overall survival; LND = lymph node dissection; PSM = propensity score matching; IPTW = inverse probability treatment weighting; NLND = no lymph node dissection; MF = mass-forming; NR = not reported; HR = hazard ratio; CI = confidence interval.

ESM_Fig_1


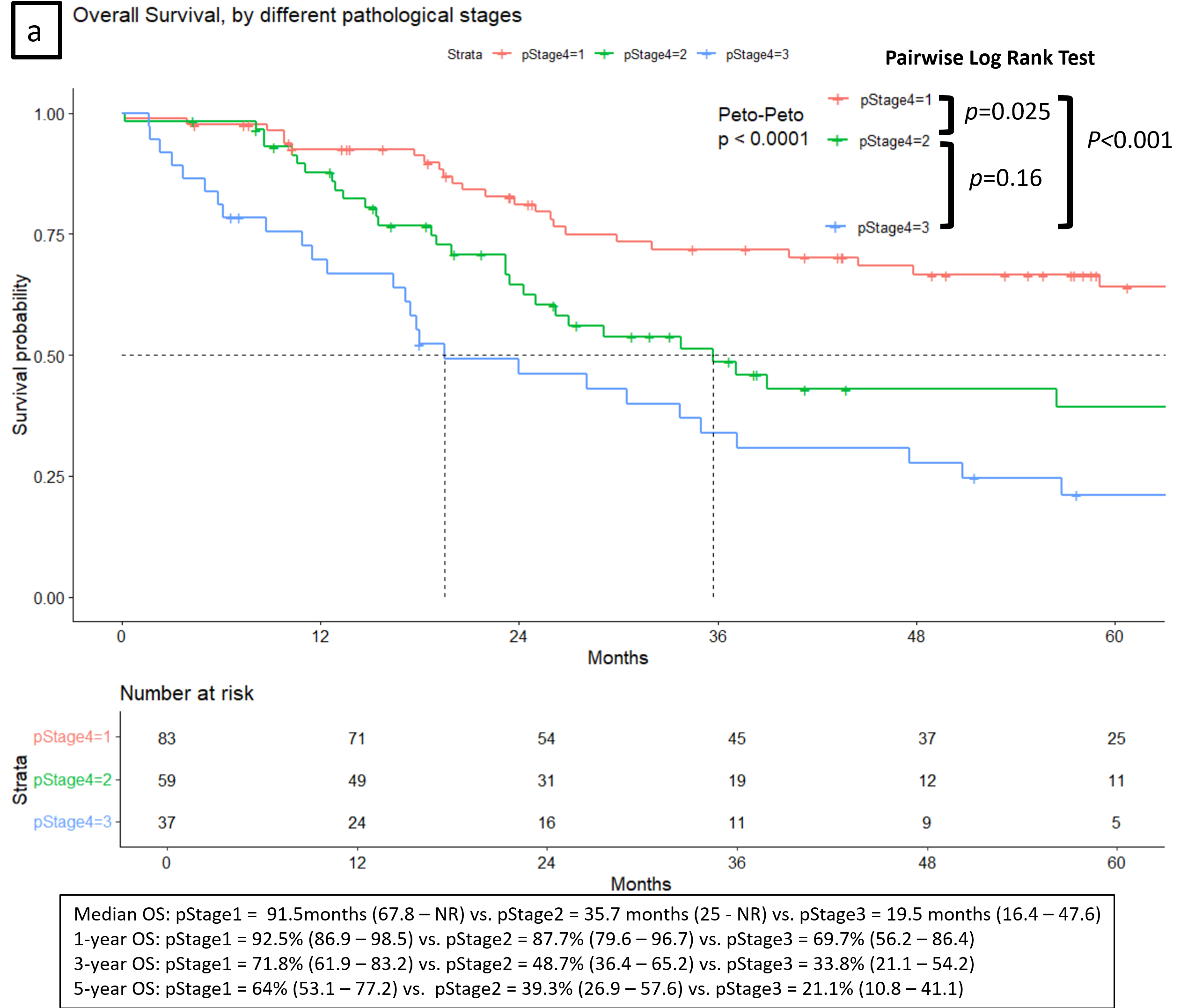


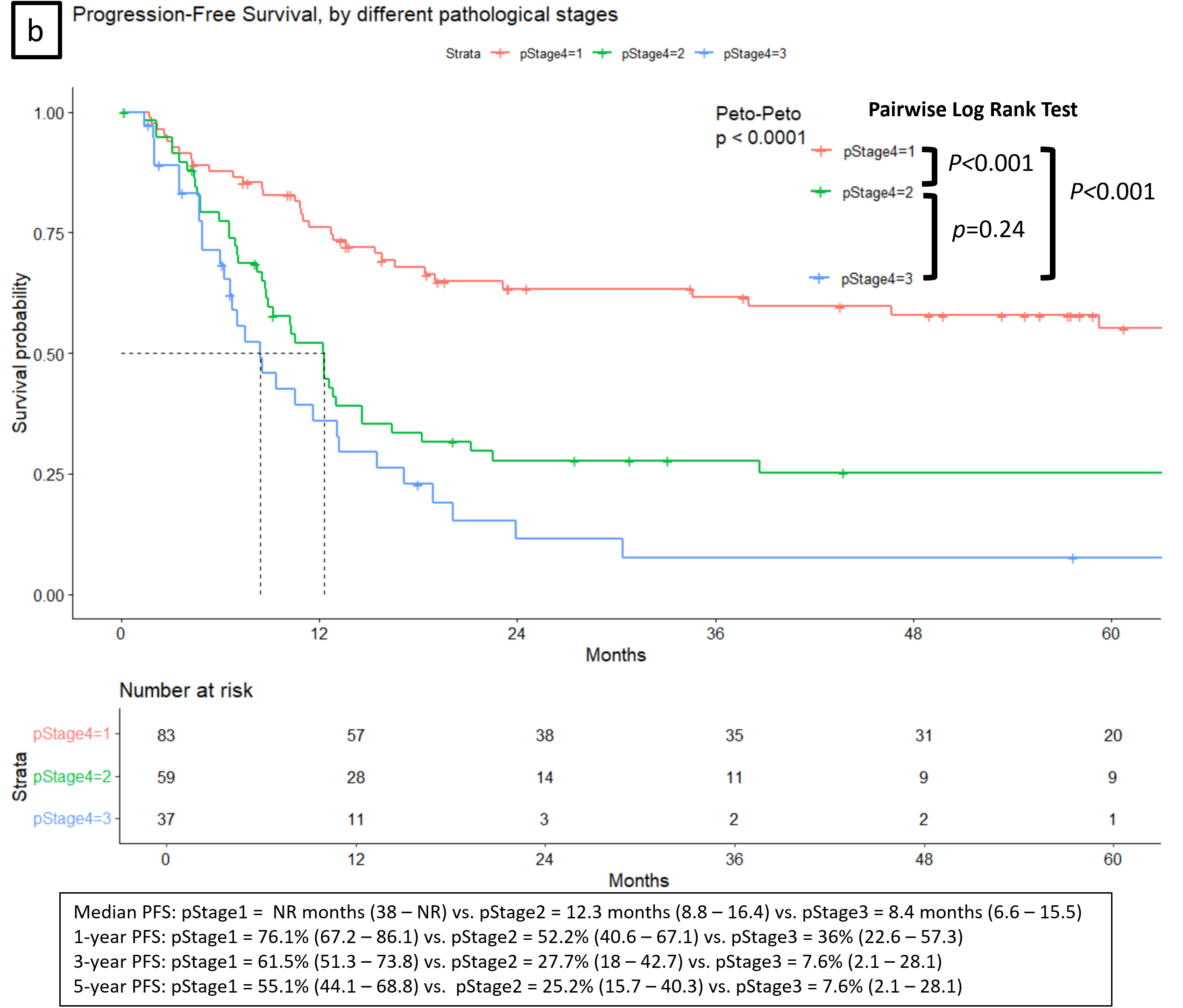


ESM_Fig_2


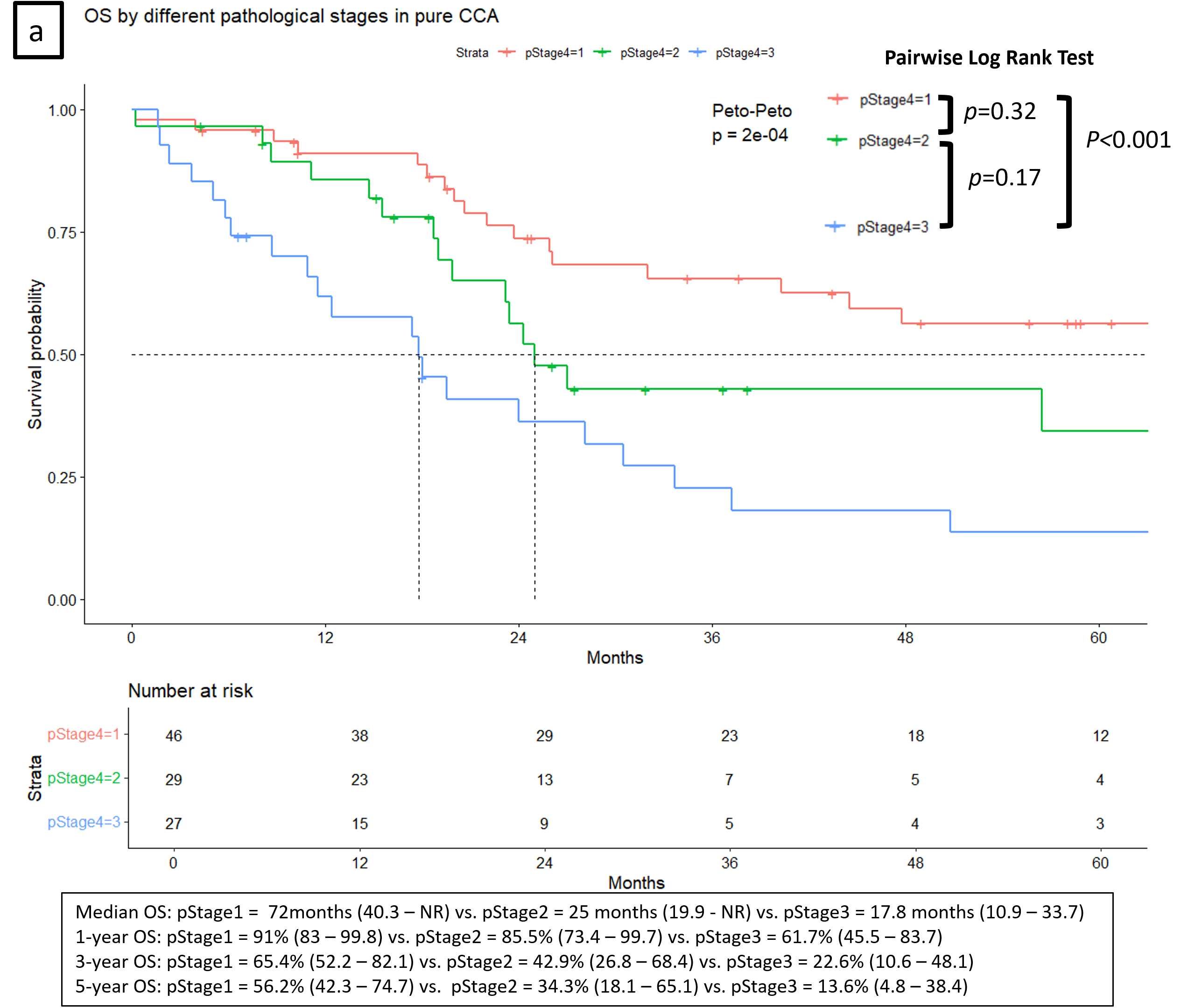


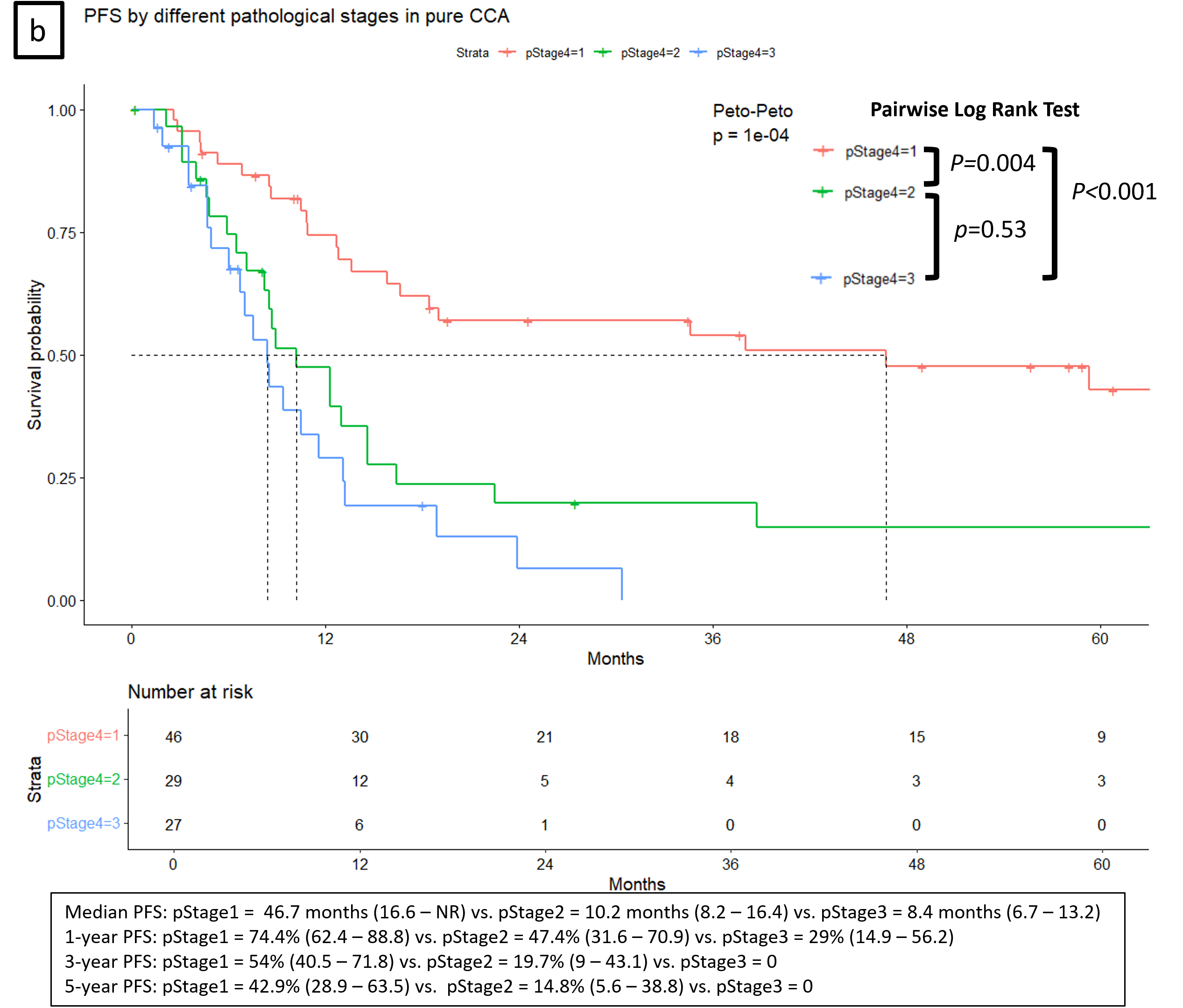


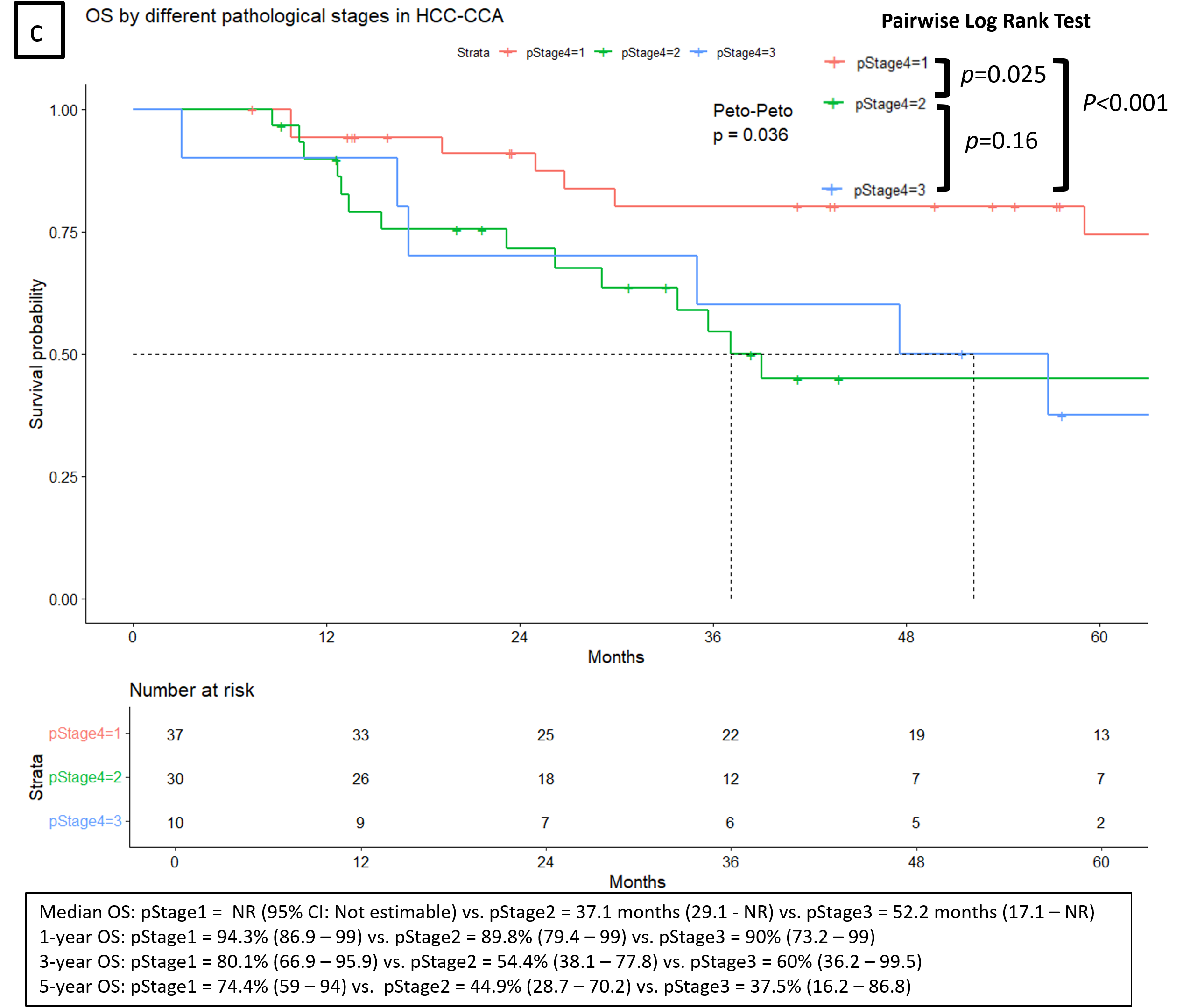


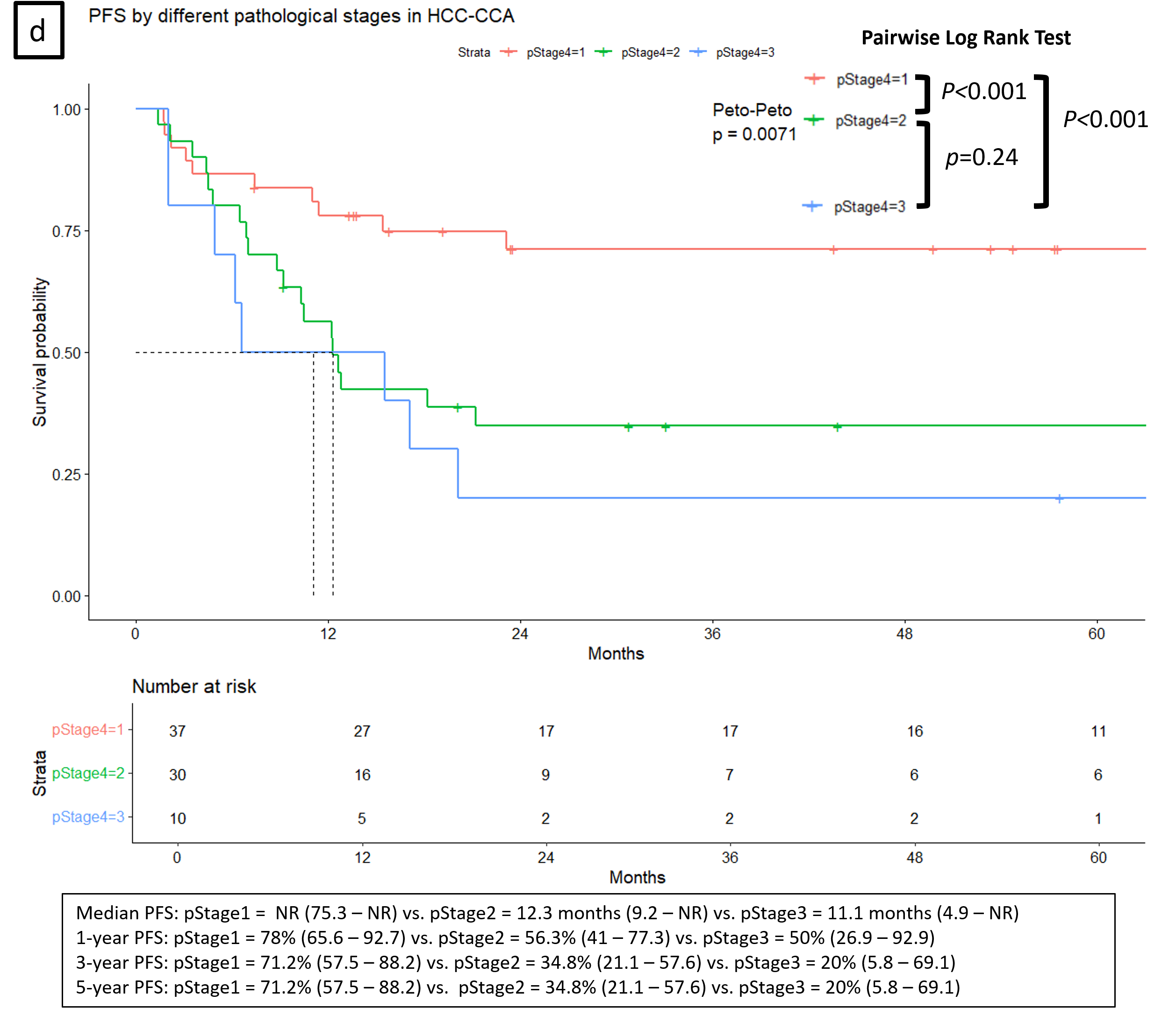


ESM_Fig_3


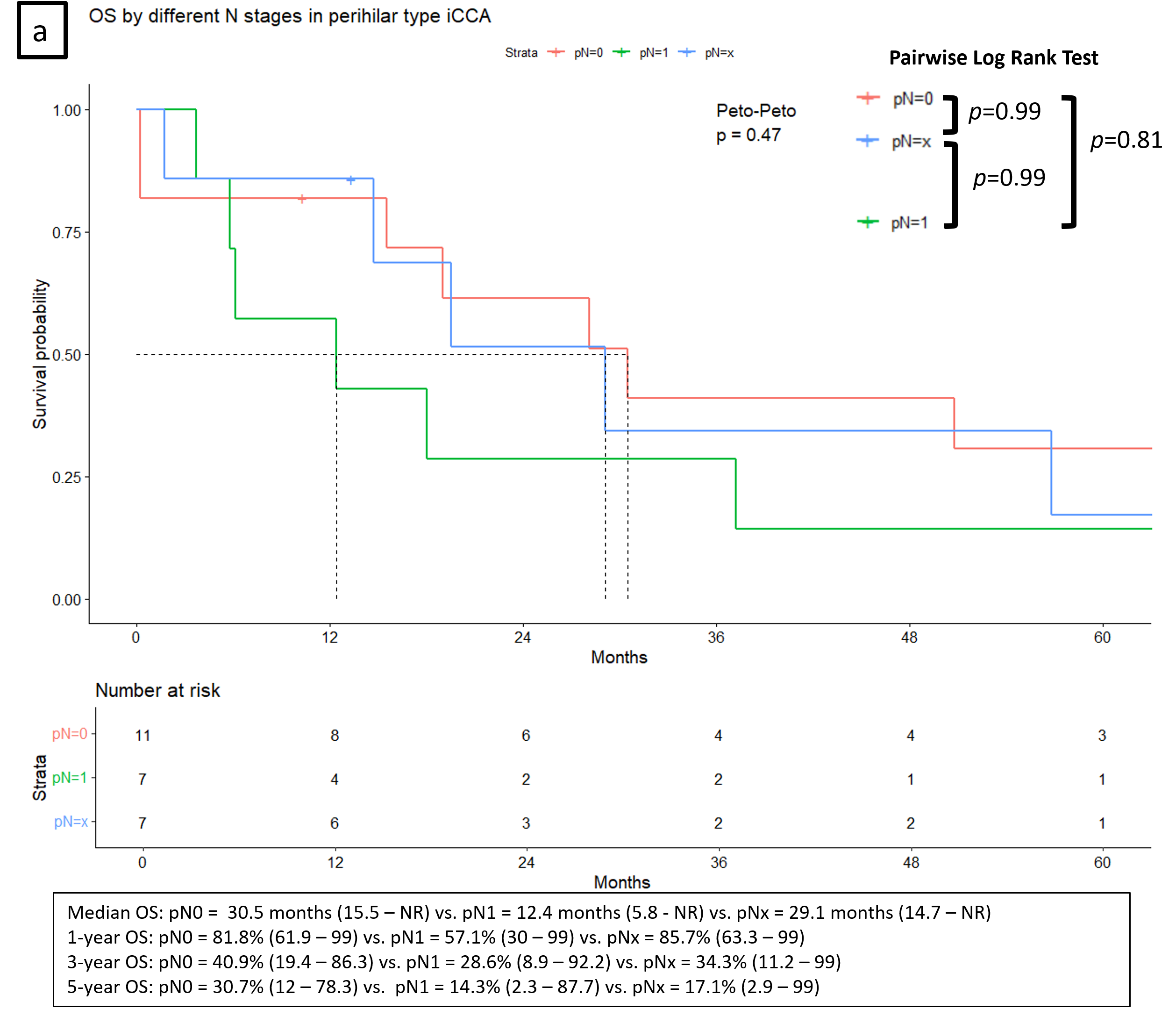


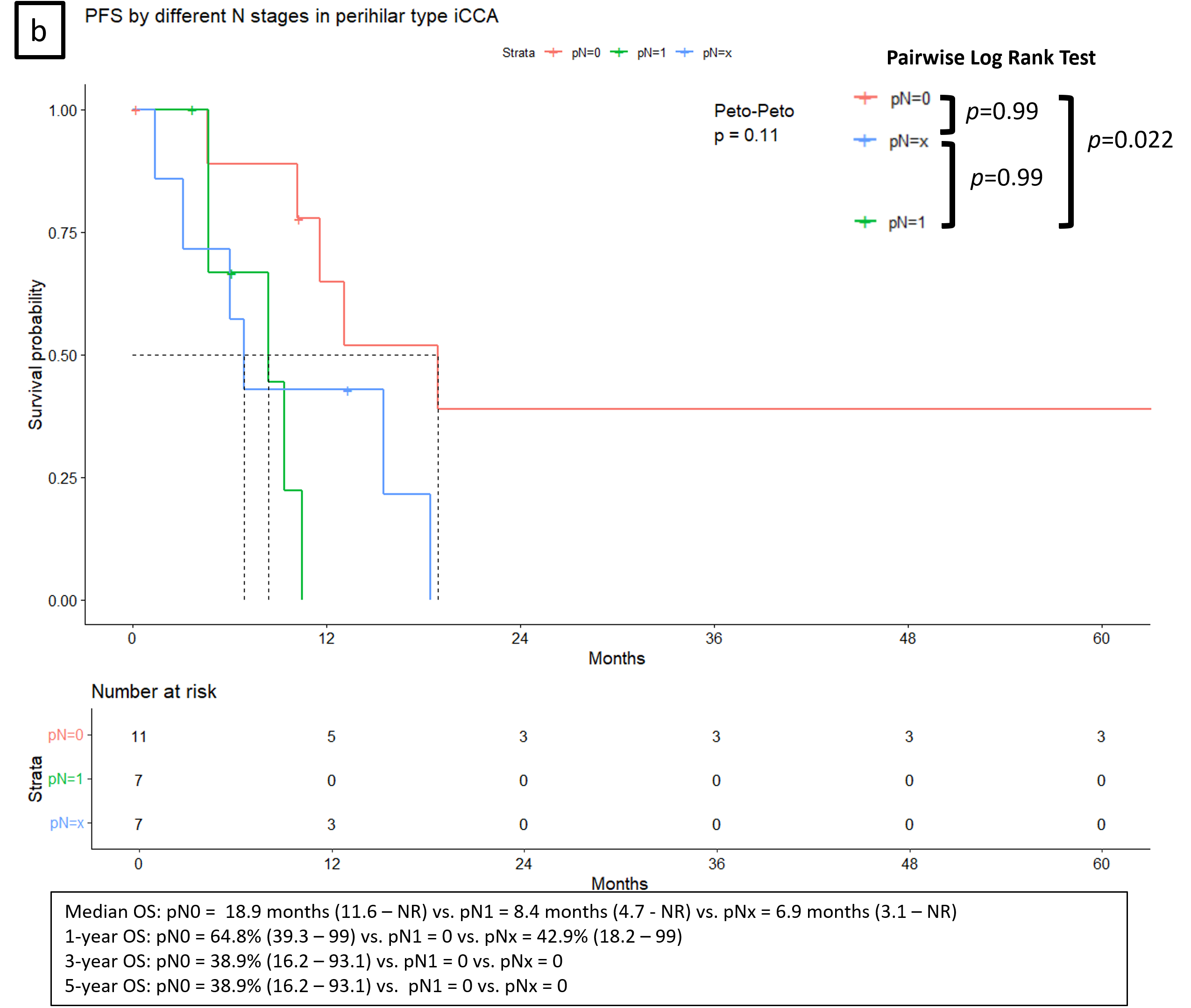


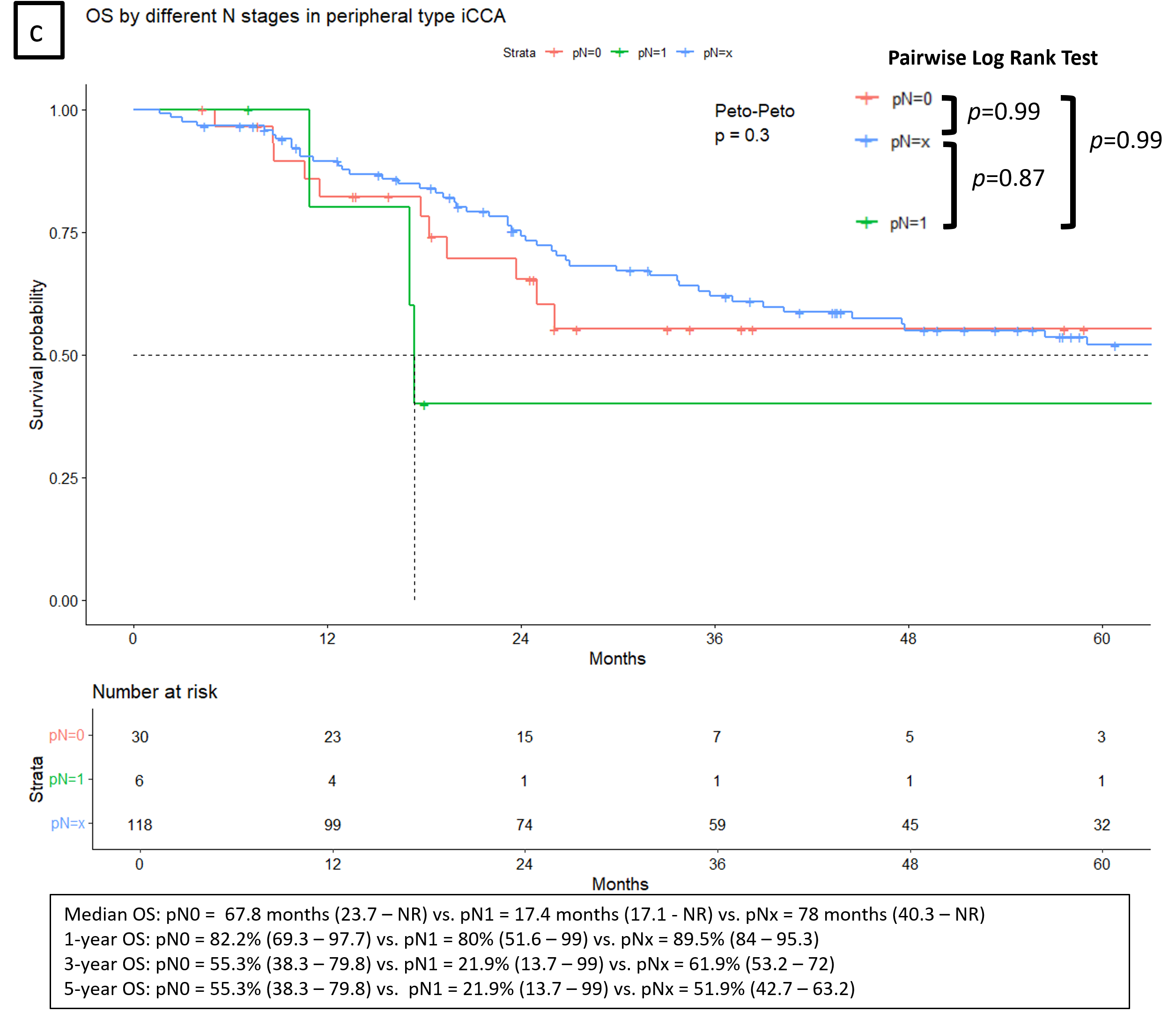


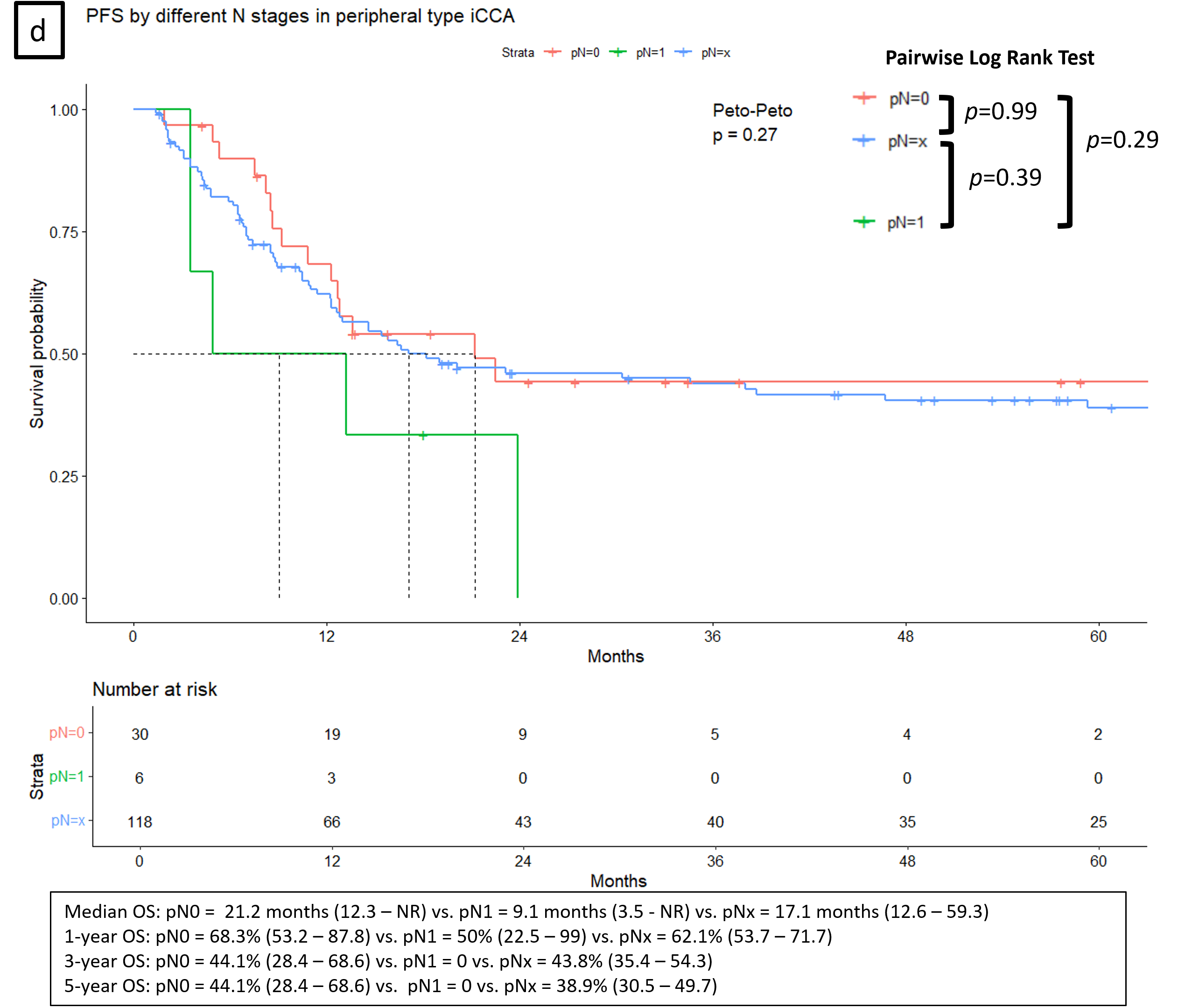

Supplement: Supplementary file 1 — Supplementary Material 1. [file 12957_2025_4034_MOESM1_ESM.docx]
